# Machine learning-based estimation of riverine nutrient concentrations and associated uncertainties caused by sampling frequencies

**DOI:** 10.1371/journal.pone.0271458

**Published:** 2022-07-13

**Authors:** Shengyue Chen, Zhenyu Zhang, Juanjuan Lin, Jinliang Huang

**Affiliations:** 1 Fujian Key Laboratory of Coastal Pollution Prevention and Control, Xiamen University, Xiamen, China; 2 Xiamen Environmental Publicity and Education Center, Xiamen, China; TDTU: Ton Duc Thang University, VIET NAM

## Abstract

Accurate and sufficient water quality data is essential for watershed management and sustainability. Machine learning models have shown great potentials for estimating water quality with the development of online sensors. However, accurate estimation is challenging because of uncertainties related to models used and data input. In this study, random forest (RF), support vector machine (SVM), and back-propagation neural network (BPNN) models are developed with three sampling frequency datasets (i.e., 4-hourly, daily, and weekly) and five conventional indicators (i.e., water temperature (WT), hydrogen ion concentration (pH), electrical conductivity (EC), dissolved oxygen (DO), and turbidity (TUR)) as surrogates to individually estimate riverine total phosphorus (TP), total nitrogen (TN), and ammonia nitrogen (NH_4_^+^-N) in a small-scale coastal watershed. The results show that the RF model outperforms the SVM and BPNN machine learning models in terms of estimative performance, which explains much of the variation in TP (79 ± 1.3%), TN (84 ± 0.9%), and NH_4_^+^-N (75 ± 1.3%), when using the 4-hourly sampling frequency dataset. The higher sampling frequency would help the RF obtain a significantly better performance for the three nutrient estimation measures (4-hourly > daily > weekly) for *R*^2^ and NSE values. WT, EC, and TUR were the three key input indicators for nutrient estimations in RF. Our study highlights the importance of high-frequency data as input to machine learning model development. The RF model is shown to be viable for riverine nutrient estimation in small-scale watersheds of important local water security.

## 1 Introduction

Waterbodies must maintain a good chemical and ecological status to protect human health and safeguard natural ecosystems. Nutrients are important indicators that affect water quality, watershed health, and biological processes [1,2]. As key constituents of riverine nutrients, high concentrations of nitrogen (N) and phosphorus (P) may lead to eutrophication and anoxia in coastal waters [3], thereby not only affecting the living environment of human beings but also the biodiversity [4]. Therefore, it is crucial to master accurate water quality data and elucidate riverine N and P dynamics for effective watershed water management, particularly for small watersheds with limited water quality monitoring but significant local water-security.

Conventional field sampling is usually conducted to examine the dynamics of N and P in fresh water [5]. However, the sampling is typically too infrequent (i.e., weekly or monthly) to fully characterize lotic nutrient conditions and to accurately estimate nutrient loading [6,7]. Additionally, the field-sampling method involves laboratory analysis to determine the concentrations of water-quality parameters, which is labor- and cost-intensive, time-consuming, and limited in terms of spatial coverage [8].

Over the past few years, with the development of online water-quality monitoring technology, the use of sensors that directly measure water quality has changed the approach to watershed research [9]. Compared to lower-frequency field sampling, higher-frequency (e.g., hourly, minutely) water quality monitoring can well capture short-term water quality dynamics and extremes. Conventional water-quality indicators, such as water temperature (WT), hydrogen ion concentration (pH), electrical conductivity (EC), dissolved oxygen (DO), and turbidity (TUR), can be monitored using probes continuously and frequently. Research methods have gradually migrated from conventional field sampling with lab analyses to online monitoring with advanced *in situ* sensors [10]. However, for many key nutrient indicators (i.e., permanganate index, Chlorophyll a, or the components of N and P), it is still difficult and/or uneconomically monitored *in situ* with high-frequency [11,12]. Moreover, there are hidden dangers and problems, such as abnormal indications caused by probe damage and sensor failure, and high maintenance costs [13,14]. The low frequency of field sampling makes it difficult to capture the instantaneous variability of water quality, and the high price of sensors prevents them from being densely deployed, thus the spatial variability of watershed water quality is difficult to capture. Insufficient water quality data caused by these problems is usually not conducive to riverine health assessment and water management.

Machine learning models have shown great potentials for estimating water quality parameters. They can solve highly nonlinear problems [15,16] and supplement mechanism models [17]. Machine learning algorithms do not consider physical processes [18], and a large number of data are often required to operate them [19]. Many studies have adopted surrogate regression to enhance the rapid generation of data input based on *in situ* measurements and to simplify resource-intensive laboratory experimentation. According to this method, the concentration of riverine nutrients can easily be estimated using alternative indicators. Researchers have used a variety of machine learning algorithms, such as neural networks (NNs; [20–22], support vector machines (SVM; [23–25], and random forest (RF; [26–29], to estimate water environment related indicators. It was found that machine learning algorithms, especially RF, have great potential and are more frequently applied for this purpose [30]. For example, different machine learning algorithms were used to compare the estimation accuracy of nutrient concentrations, and the results showed that RF was significantly more accurate than other conventional algorithms when estimating all six levels of water quality (I, II, III, IV, V, and worse than V [WV]), which are based on the National Environmental Quality Standards for surface water of China (GB3838-2002) [31]. The RF, gradient boost regression, and AdaBoost regression have been used to simulate the daily suspended sediment load in the Mississippi River, and the result show that RF is slightly ahead in prediction performance [32].

It is well known that uncertainty is inherent in model development [33]. Many studies were devoted to exploring the causes of uncertainty in machine learning models to improve estimation accuracy [34,35]. Sharafati et al. [35] used a Monte Carlo simulation model to quantify estimation uncertainties. The results showed that the model structures were more influential than the input indicators for estimating effluent quality parameters. Noori et al. [36] used the percentage of observed data bracketed by 95% predicted uncertainties (95PPU) and the bandwidth of 95% confidence intervals (*d*-factor) to analyze the uncertainties brought by SVM hyperparameters. They found that the model was more sensitive to the capacity parameter (C) than to kernel parameters (Gamma) and error tolerance (Epsilon). Not just hyperparameter and model structure, data input associated with different sampling frequencies might also induce uncertainties and influence estimation accuracy [37]. Derot et al. [2] demonstrated that the different sampling frequency datasets directly impact the forecast performance of an RF model. According to their findings, the accuracy of phytoplankton bloom forecasts for a 20-min time step was higher than that of the 1-day time step. It appears from these studies that there are many kinds of factors that affect the estimation accuracy and associated uncertainty. Among those factors, the model uncertainty caused by the frequency of data input might be more worthy of discussion with the increasing popularity of automatic monitoring sensors.

The estimation accuracy of nutrient concentration depends not only on the model structure but also on the amount and type of data input [31]. Many researchers used multiple types of indicator inputs for estimation [38] or indicators having high correlation with the substances to be tested as inputs. Some even used one nutrient to estimate another type of nutrient. Although desired estimation results can be achieved, these methods are difficult to implement in reality because some of the input indicators (chemical oxygen demand, nitrate, and nitrite, etc.) are not readily available in a high temporal resolution [39]. Therefore, it is crucial to develop a convenient as well as accurately model of nutrient concentration estimation that the input indicators are easier available.

Despite that many studies have been focused on machine learning in different fields, few researches have combined machine learning methods with high-frequency monitoring data and evaluate model uncertainty caused by frequency of data input. To develop a model that can estimate riverine nutrient (total phosphorus [TP], total nitrogen [TN], and ammonia nitrogen [NH_4_^+^-N]) concentrations easily and accurately, as well as evaluate the uncertainty caused by the sampling frequency, thus helpful to water management in a small-scale watershed, we developed an RF model using datasets of only five monitoring water-quality indicators (i.e., WT, pH, EC, DO, and TUR) from the unique online multi-parameter water-quality sensor located in the outlet of the watershed (sensor type can be seen in S1 Text, Supporting information). Concurrently, we constructed an SVM and a back-propagation neural network (BPNN) for performance comparison. All these three machine learning models are widely used, and with well estimation accuracy. Specifically, the main objectives of this study are (1) to compare the estimative performance of different machine learning models for riverine nutrient concentrations, and (2) to evaluate the accuracies and uncertainties of the models with datasets of different sampling frequencies (i.e., 4-hourly, daily, and weekly). The findings of this study would be helpful to easily estimating riverine nutrient concentrations in small-scale watersheds and evaluating the contributions of high-frequency data to estimation accuracy. The proposed model strategy can be used in other small-scale watersheds with scarce data on nutrients but easily available and high frequency chemical/physical indicators to improve the efficiency of machine learning models used for water-quality estimation.

## 2 Data and methodology

**Fig 1:**
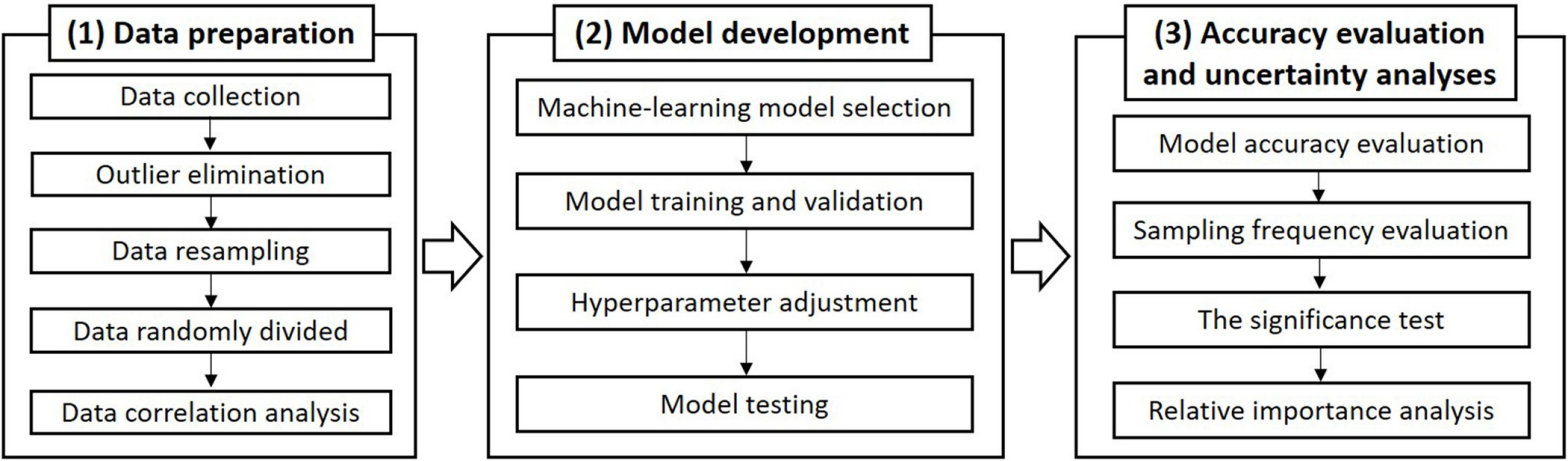
Flowchart of the proposed methodology.

Herein, a data-driven methodology based on machine learning is proposed to measure uncertainties due to three different sampling frequencies while estimating the riverine nutrient concentrations. As shown in Fig 1, this technique route comprises three components: (1) data preparation, (2) model development, and (3) accuracy and uncertainty analyses. The methods and formulations involved are described exhaustively in the following sections.

### 2.1 Data preparation

**Fig 2:**
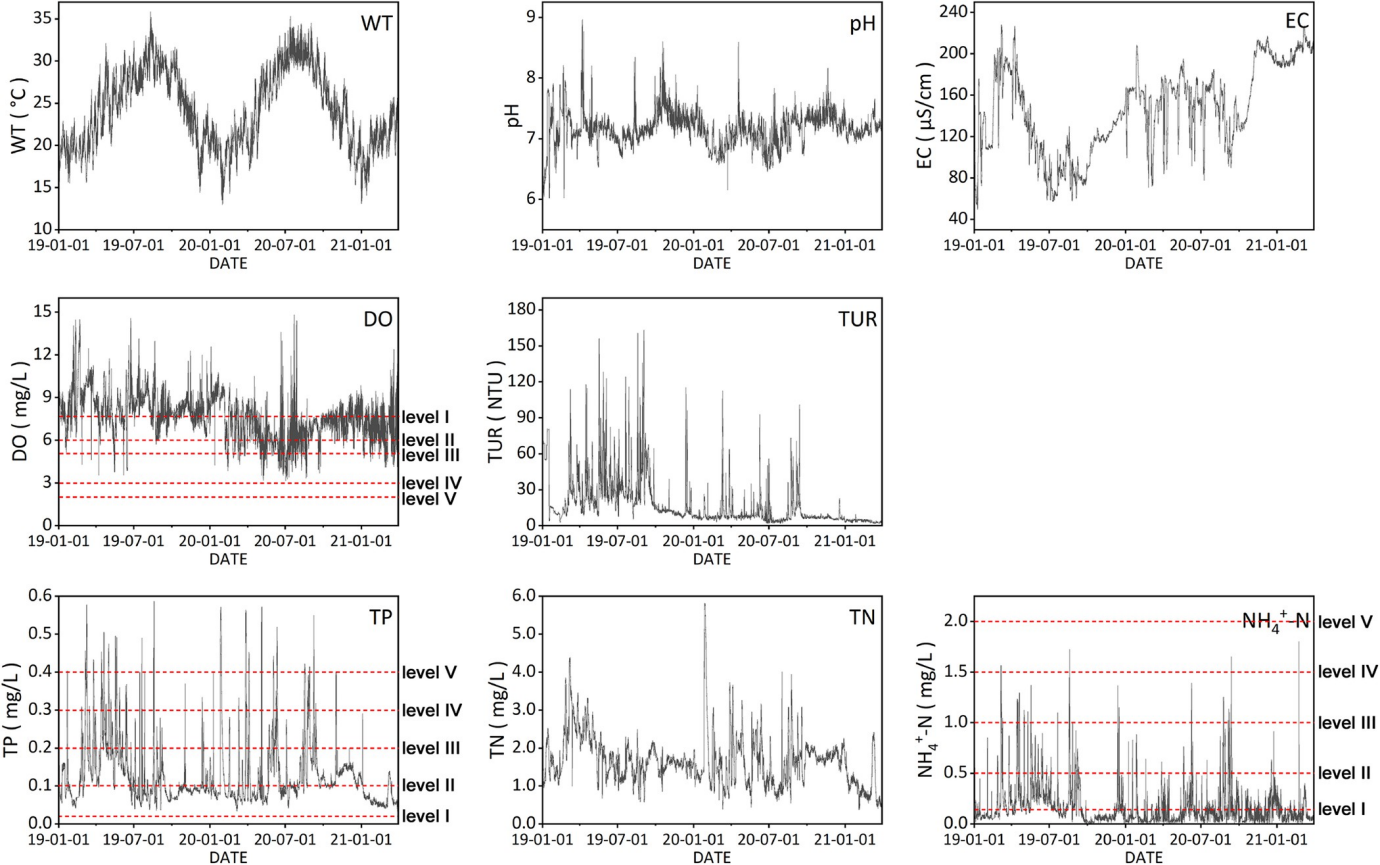
The 4-hourly variation of sensor readings from January, 2019, to March, 2021, for the water quality indicators in the outlet of the Aitoutan (ATT) watershed. The red dotted lines represent the boundary of environmental quality standards for surface water in China. The water quality levels gradually deteriorate from level Ⅰ to level Ⅴ and the value of indicators exceeding the level Ⅴ is defined as “worse than Ⅴ”. For DO, the higher value represents the better water quality level, and for TP and NH_4_^+^-N, the higher value represents the worse water quality level.

The Aitoutan (ATT) watershed is located in Tong’an District, Xiamen, China. Since China launched environmental regulations (e.g., “River Chief”) in 2016, water quality in the ATT watershed has been significantly improved. In recent years, the main pollutant faced by the watershed is TP, and the sensor-monitoring data at the outlet of the watershed shows that the concentration of TP frequently exceeds the level III based on National Environmental Quality Standards for surface water of China (higher than 0.2 mg/L) (Fig 2). Thus, water quality is still a concern for local governments.

The data of the monitoring site in the study area was acquired by sensors in the surface water, and the other monitoring indicators except nutrients are used as the input indicators of the machine learning models. The dataset in this study comprises five physical/chemical indicators used as inputs of machine learning models, namely WT, pH, EC, DO, and TUR, and three nutrients being estimated, namely TP, TN, and NH_4_^+^-N, which covers the period from January 1, 2019, to March 31, 2021, and was provided by the Xiamen Environmental Publicity and Education Center (specific information can be seen in S6 Text, Supporting information). The outliers (each water quality indicator value lower than/equal to 0 and the null value) were eliminated from this dataset. This dataset has a temporal resolution of four hours, which denotes that the water-quality indicators were automatically monitored by an interval of four hours from midnight daily. We resampled this 4-hourly frequency monitoring dataset to mimic both daily and weekly monitoring schemes. The water-quality indicators at 8 a.m. each day were extracted as a daily dataset, and the indicators at 8 a.m. each Monday were extracted as a weekly dataset. The three datasets of sampling frequency scenarios have the same temporal span. The 4-hourly dataset includes 4,209 samples of water quality indicators (five physical/chemical indicators and three nutrients as described above), whereas the daily dataset includes 803 samples; the weekly dataset has 115 samples. The samples in each dataset are at the same time step, that is, there is no time lag in the input samples in this study.

As summarized in Table 1, the descriptive statistics of these five input indicators and three nutrients with the 4-hourly frequency showed that the indicators having the highest coefficients of variation (CV) were TUR and NH_4_^+^-N, and the most stable indicator was pH. The CVs of WT and DO as well as TUR and NH_4_^+^-N were similar in pairs. The standard deviation (SD) was used to measure the data deviation from the mean value. CV is the mean normalized SD, and it represents the statistical dispersion of data. Before model development, the input indicators and nutrients of training set of the 4-hourly dataset will undergo Spearman’s test of rank correlation to determine whether the correlation between the five input indicators and nutrients are too high.

$$SD=\sqrt{\frac{1}{N-1}\sum_{i=1}^{N}{\left(O_{i}-\bar{O}\right)}^{2}}$$ (1)

$$CV\%=\frac{SD}{\bar{O}}\times 100$$ (2)

where *n* is the number of input samples, *O*_*i*_ is the observations, and $\bar{O}$ represents the mean values of the observations.

**Table 1:** Descriptive statistics of input indicators and output nutrients from the monitoring site located in the outlet of Aitoutan (ATT) watershed.

Parameter	Max	Min	Mean	SD	CV (%)
WT (°C)	35.83	13.00	24.27	4.58	18.88
pH	8.96	6.03	7.16	0.28	3.92
EC (μS/cm)	227.95	49.80	146.44	42.24	28.84
DO (mg/L)	14.79	3.17	7.66	1.56	20.40
TUR (NTU)	162.97	1.90	16.70	17.94	107.41
TP (mg/L)	0.59	0.03	0.13	0.08	64.82
TN (mg/L)	5.81	0.38	1.66	0.69	41.28
NH_4_^+^-N (mg/L)	1.92	0.01	0.16	0.19	113.55

Notes: CV = “coefficient of variation”; SD = “standard deviation”.

### 2.2 Model development

MATLAB 2019b was used in this study to develop the RF, BPNN, and SVM model. To prevent overfitting of the models and ensure the generalization ability of the model, 80% of the dataset was randomly selected as the training set first, and the remaining 20% was selected as the testing set. The training set was then divided into a training-validation set based on a 10-fold cross-validation [40,41]. In this study, the training set was used for model fitting, the validation set was used to pick the optimal hyperparameter combination, both training set and validation set here were in 10-fold cross-validation phase, and we determined the optimal hyperparameters by the average of the statistical metrics of the validation set under 10-fold cross-validation. Then we iterated the optimal hyperparameter combination to three machine learning models, fit the models with the initially divided training set, and test the generalization ability of the models in the testing set. We selected the optimal model from three machine learning models (Section 3.2) and evaluate the estimation accuracy and uncertainty of the selected model with three sampling frequency scenarios (Section 3.3).

### 2.3 Accuracy evaluation and uncertainty analysis

The three machine learning models were evaluated for the estimation accuracy of cross-validation step under the 4-hourly frequency scenario, and the model with the best performance of validation set would be selected for the next phase (accuracy and uncertainty analysis due to different sampling frequencies). Several statistical metrics were selected to evaluate the estimation accuracy and uncertainty of the models proposed in this study. The coefficient of determination (*R*^2^), Nash-Sutcliffe efficiency (NSE), root mean squared error (RMSE), and mean absolute error (MAE) were used to assess the goodness of fit between the observed nutrient concentrations and those estimated by three models.

$$R^{2}=\frac{{\left[\sum_{i=1}^{n}\left(O_{i}-\bar{O}\right)(P_{i}-\bar{P})\right]}^{2}}{\sum_{i=1}^{n}{\left(O_{i}-\bar{O}\right)}^{2}\sum_{i=1}^{n}{\left(P_{i}-\bar{P}\right)}^{2}}$$ (3)

$$NSE=1-\frac{\sum_{i=1}^{n}{\left(O_{i}-P_{i}\right)}^{2}}{\sum_{i=1}^{n}{\left(O_{i}-\bar{O}\right)}^{2}}$$ (4)

$$RMSE=\sqrt{\frac{1}{n}\sum_{1}^{n}{\left(O_{i}-P_{i}\right)}^{2}}$$ (5)

$$MAE=\frac{1}{n}\sum_{1}^{n}\left|\left(O_{i}-P_{i}\right)\right|$$ (6)

where *n* is the samples of training/validation/test sets in 4-hourly/daily/weekly frequency scenario; *O*_*i*_ and *P*_*i*_ are respectively the observations and model estimations for each set; $\bar{O}$ and $\bar{P}$ respectively represent the mean values of the observations and model estimations for each set. Usually, *R*^2^ and NSE values closer to 1 while RMSE and MAE values closer to 0 denote higher accuracy.

In this phase, to evaluate the estimation accuracy and uncertainty caused by sampling frequencies, we first selected the model with the highest estimation accuracy from the three machine learning models. We resampled the 4-hourly dataset to extract daily and weekly sets according to the pattern in Section 2.1 and nine scenarios (i.e., three nutrients × three sampling frequencies) were designed. The testing set of the 4-hourly scenario has 842 samples (20% previously split from the 4-hourly dataset). The datasets of daily and weekly sampling frequency scenarios were all used as training-validation sets for their respective models based on the k-fold cross-validation. In order to equally evaluate and compare the impact of three sampling frequency scenarios on the estimation accuracy of RF, we chose the testing set of 4-hourly scenario, and from it we randomly selected 20% of the total samples of daily/weekly scenario as the testing sets for the daily/weekly scenarios. Therefore, the training set of the daily scenario has 803 samples and the testing set has 161 samples; the training set of the weekly scenario has 115 samples and the testing set has 23 samples. We performed 30 replicate estimations under this dataset division, and evaluated the model accuracies and uncertainties in testing sets under three sampling frequency scenarios. The statistical metrics for estimation accuracies of testing sets were used for the one-way analysis of variance (ANOVA) test to evaluate whether there is a significant difference in the estimation accuracy between the three sampling frequencies.

One of the main advantages of RF is that it can assess the importance of the input indicators used in the modeling processes [42]. It is vital to identify some key water indicators when model developing. To further optimize the machine learning model and improve the comprehensive management of watersheds, the RF model was selected to analyze the relative importance of the input indicators. For each nutrient, the weights and relative importance of the input indicators were ranked and analyzed. The calculation method of the importance of each indicator in RF is as follows: (1) For each decision tree in the RF model, the out-of-bag (OOB) data are used to calculate OOB error, denoted as OOBE1. (2) Redistribute all the original N samples of each indicator through permutation, the OOB error is calculated again and recorded as OOBE2. (3) Assuming that there are N trees in the RF model, the relative importance for each indicator can be shown in Eq (7):

$${RI}_{i}=\frac{\sum_{1}^{n}\left[\frac{\sum_{1}^{N}({OOBE2}_{i}-{OOBE1}_{i})}{N}\right]}{n}$$ (7)

where *RI*_*i*_ refers to the relative importance of each indicator, *N* denotes the amounts of tree of RF model, and *n* is the number of indicators.

## 3 Results

### 3.1 Correlation analysis of water quality indicators

**Fig 3:**
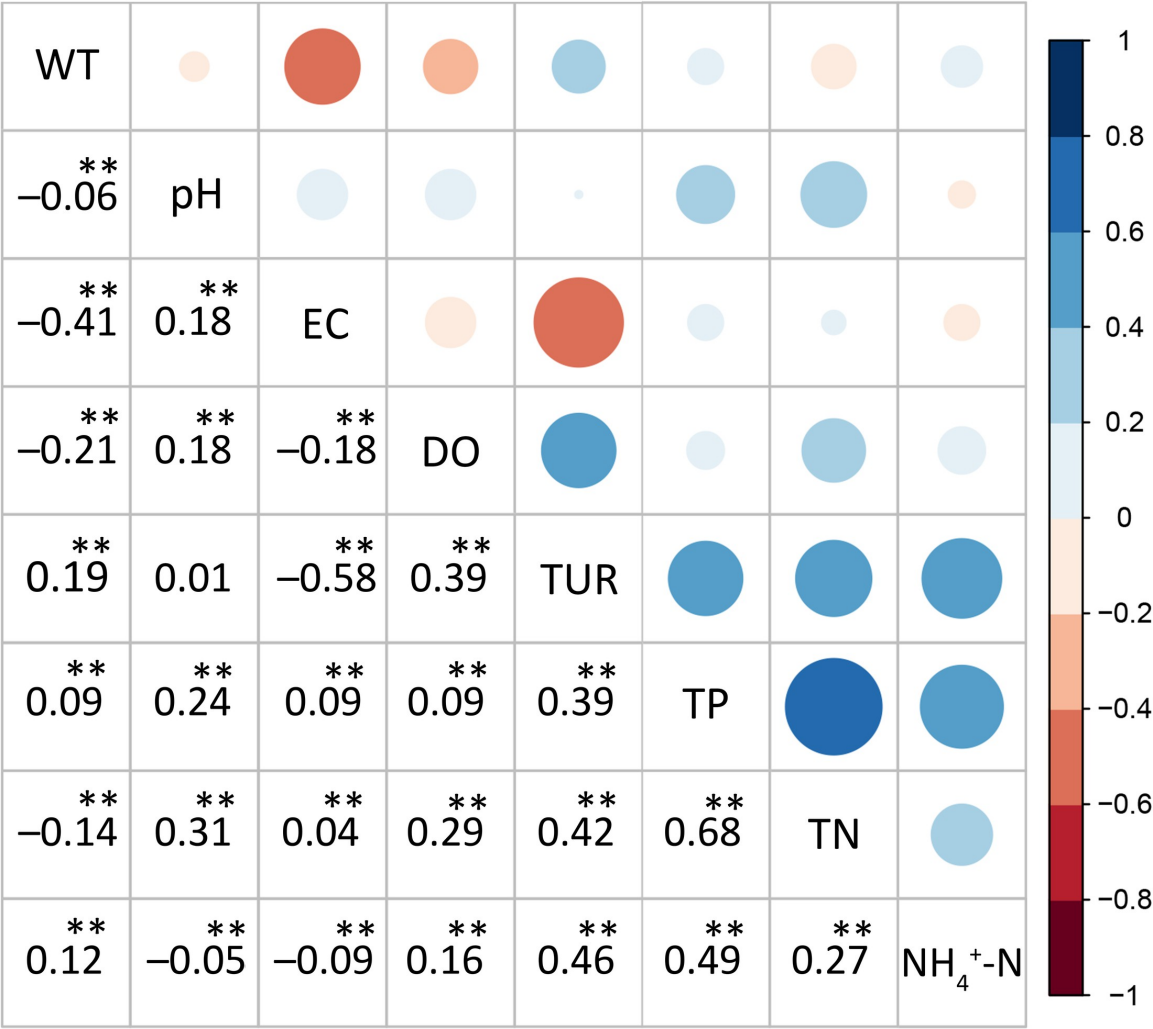
Correlation analysis for the input and output indicators. The statistical significance of rank correlations is denoted by asterisks for *p* < 0.05 (*) and *p* < 0.01 (**) (lower left). The different sizes and colors of circles represent the strength of the correlation between the indicators (upper right).

Based on Spearman’s test of rank correlation, there was a large number of high statistically- significance (i.e., p < 0.01) among the nutrients and input indicators (Fig 3). As shown in this figure, TUR is strongly positively correlated with all three nutrients, DO is positively correlated with all three nutrients, EC is weakly positively correlated with TP and TN and weakly negatively correlated with TN, pH is positively correlated with TP and TN and negatively correlated with NH_4_^+^-N, and WT is weakly positively correlated with TP and NH_4_^+^-N and negatively correlated with TN. The correlation analysis of the nutrient concentrations showed that TP was strongly positively correlated with TN and NH_4_^+^-N, while the correlation between TN and NH_4_^+^-N was relatively low.

### 3.2 Evaluation of estimation accuracy among three machine learning models

The sampling frequency of data we used in this phase was the 4-hourly scenario, and the three models used the same division rules for the dataset. Different machine learning models using the same dataset for estimation may have different performances. The hyperparameter selections of three machine learning models can be found in S2, S3, and S4 Text in Supporting information. The performances of testing set can be seen in Table 2. For each nutrient, the *R*^2^ and NSE obtained by RF are higher than SVM and BPNN, whereas the RMSE and MAE of RF are the lowest among three models.

**Table 2:** Comparison of the average estimation accuracy of the three machine-learning models (4-hourly frequency, testing step, n = 842).

Model	Statistical metric	Nutrient		
		TP	TN	NH_4_^+^-N
RF	*R* ^2^	0.801	0.859	0.759
	NSE	0.785	0.853	0.748
	RMSE	0.039	0.284	0.087
	MAE	0.024	0.189	0.057
SVM	*R* ^2^	0.737	0.811	0.720
	NSE	0.734	0.810	0.717
	RMSE	0.044	0.316	0.095
	MAE	0.025	0.219	0.054
BPNN	*R* ^2^	0.668	0.757	0.616
	NSE	0.666	0.754	0.602
	RMSE	0.049	0.361	0.113
	MAE	0.031	0.268	0.072

**Fig 4:**
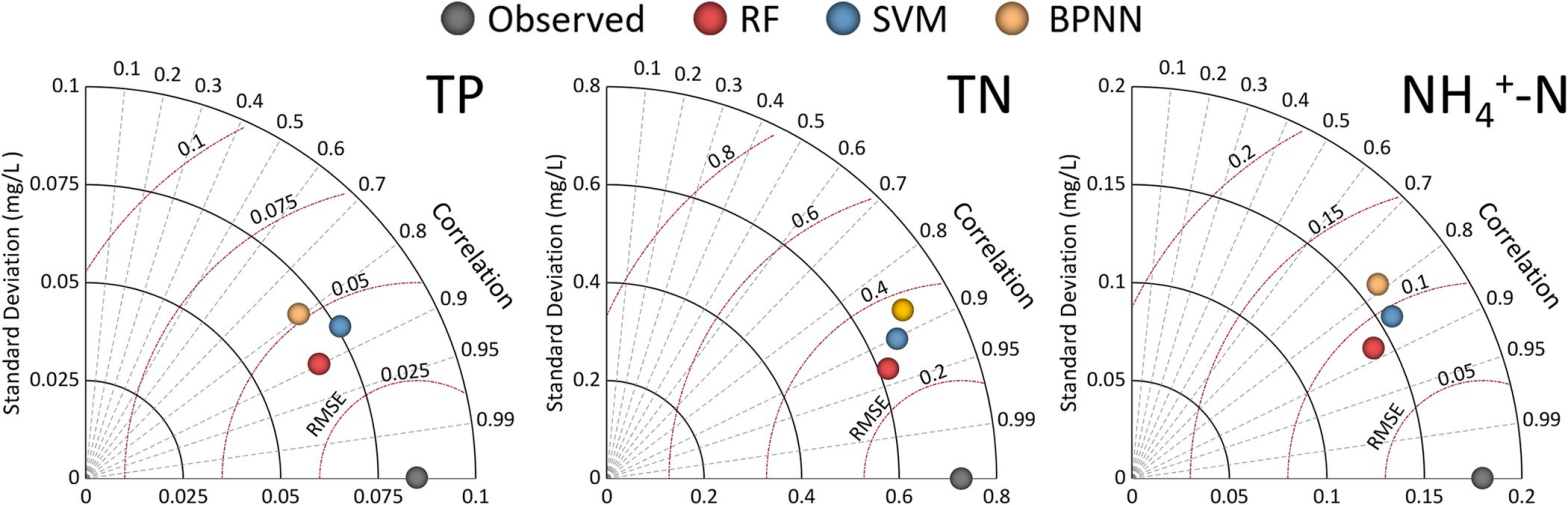
Comparison of the models’ performances by Taylor diagrams. RF = “random forest”; SVM = “support vector machine”; BPNN = “back-propagation neural network”; TP = “total phosphorous”; TN = “total nitrogen”; and NH_4_^+^-N = “ammonia-nitrogen”.

This study uses Taylor diagrams to make visual comparisons of results obtained by the three models (Fig 4). Model performance is represented by a point, where the most accurate model has the closest distance to the point of observation, which is shown by the dark-grey point in the diagrams. Based on the principle of the Taylor diagram (i.e., correlation, standard deviation, and RMSE), the RF model has higher correlations with observed nutrient concentrations and a lower RMSE compared with the two other models. Fig 4 confirms that the RF model provides the highest accuracy when estimating TP, TN, and NH_4_^+^-N concentrations. Moreover, the BPNN model has the weakest performance compared with the other models.

### 3.3 Evaluation of model accuracy with different sampling frequency scenarios

We chose the RF model that had the highest *R*^2^ and NSE and the lowest RMSE and MAE values in testing step under 4-hourly scenario (Table 2) for subsequent use. The hyperparameter selections of RF were consistent with Section 3.2. We performed 30 replicate estimations for each of nine scenarios (i.e., three nutrients × three sampling frequencies) as described in Section 2.3. The mean results of testing phase are presented in Table 3. Among them, the rank of *R*^2^ and NSE values of the RF model under three sampling frequency scenarios is 4-hourly > daily > weekly. When the sampling frequency was increased from weekly to 4-hourly, the *R*^2^ and NSE obtained by the RF model is greatly improved (TP 30%, TN 30%, and NH_4_^+^-N 25% for *R*^2^; TP 36%, TN 31%, and NH_4_^+^-N 34% for NSE). Regarding RMSE and MAE, there is no such pattern. Among the average estimation results of the RF model with three sampling frequency scenarios, the values of RMSE and MAE do not change much compared with *R*^2^ and NSE.

**Table 3:** Comparison of the average estimation accuracy of the RF model with three sampling frequencies (testing step).

Sampling frequency	Statistical metric	Nutrient		
		TP	TN	NH_4_^+^-N
Four-hourly (n = 842)	*R* ^2^	0.785	0.840	0.749
	NSE	0.781	0.837	0.747
	RMSE	0.038	0.285	0.087
	MAE	0.023	0.187	0.057
Daily (n = 161)	*R* ^2^	0.692	0.761	0.676
	NSE	0.667	0.735	0.668
	RMSE	0.041	0.301	0.093
	MAE	0.026	0.196	0.065
Weekly (n = 23)	*R* ^2^	0.602	0.658	0.598
	NSE	0.574	0.639	0.559
	RMSE	0.044	0.318	0.101
	MAE	0.029	0.201	0.068

**Fig 5:**
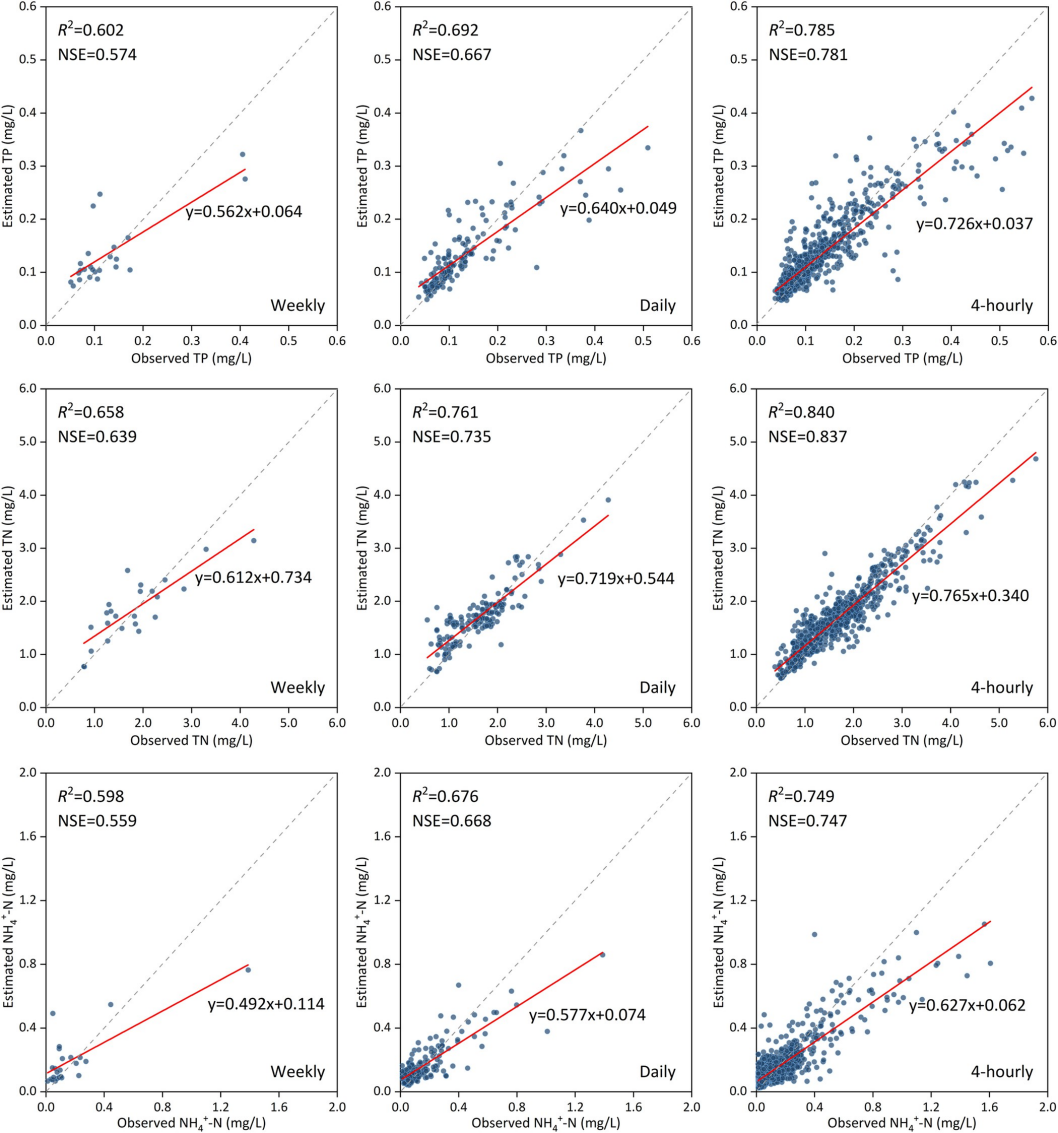
Scatterplots of the observations and average estimations with three sampling frequency scenarios. The x-axis represents the observations while the y-axis represents the estimations. The grey dashed line represents the 1:1 fitted line of observations and estimations under ideal conditions. The red line represents the fitted line of observations and estimations in actual situation.

The scatterplots can characterize the relationship between observed values (i.e., three nutrients with three sampling frequencies) and the average estimation results of the RF model in the testing phase (Fig 5). Results show that as the sampling frequency increases, the slope of the fitted line between the estimated value and the observed value constantly approach 45° (slope = 1), which also results in the increase of model estimation accuracy. For different nutrients, the slope of the fitted line can also prove the rank of model estimation accuracies (TN > TP > NH_4_^+^-N). When the actual values (i.e., observed nutrient concentrations) are lower than half of their maximum values, overestimation and underestimation by RF exist simultaneously; however, when the actual values are higher than half of their maximum value, the RF tends to underestimate, which is more obvious at the peak of observations. The error between observations and estimations at the peak (especially underestimation) may be the main reason to affect the slope.

**Fig 6:**
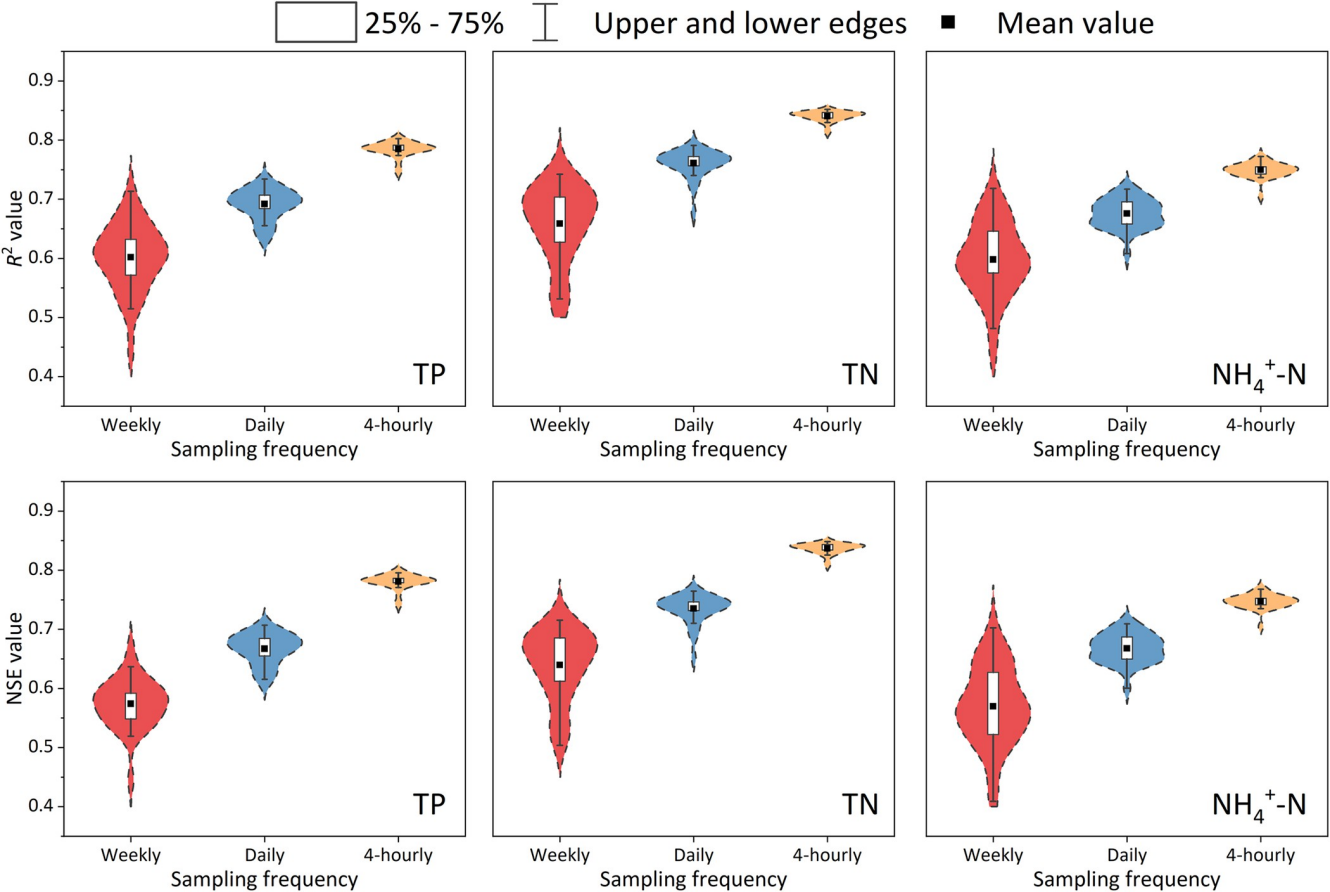
Estimated *R*^2^ and Nash-Sutcliffe efficiency (NSE) values for the random-forest (RF) model under different sampling frequency scenarios. The width of the violin shape indicates the frequency at which *R*^2^ and NSE appear at this value.

The 30 replicate estimation results under various scenarios are also displayed in a violin plot (Fig 6). This representation not only shows the quantile, but it also provides the kernel density curve of the data. In view of the results in which the variation of RMSE and MAE are minimal compared with *R*^2^ and NSE, we only chose *R*^2^ and NSE to evaluate the performance of different sampling frequencies. As shown in Fig 6, for all nutrients, the mean values of *R*^2^ and NSE after 30 RF estimations under the 4-hourly frequency are higher than those of the daily frequency. The weekly one has the lowest *R*^2^ and NSE. It can be observed from the inside boxes that *R*^2^ and NSE values obtained by RF with via 4-hourly sampling frequency scenario have the smallest changes under each scenario. Thus, they maintain a high level. For comparison, the estimation accuracy of RF under the weekly scenario fluctuates greatly, and the high (e.g., *R*^2^ and NSE about 0.7) and the low (*R*^2^ and NSE about 0.4) accuracies appear at the same time. Hence, the mean values are the lowest in the end. Regarding the comparison of estimation accuracies among the different nutrients, driven by the same sampling frequency data input, TN always obtains the highest *R*^2^ and NSE values, whereas NH_4_^+^-N is always the lowest.

An ANOVA test was performed to confirm whether the uses of dataset with different sampling frequencies cause significant differences in the estimation accuracy of the RF model. The results are presented in Table 4. For each group (one nutrient × one statistical metrics), the differences of three sampling frequencies are significant. The estimation accuracy of the RF model under the 4-hourly frequency is significantly better than that of the daily frequency, and the daily frequency is also significantly better than the weekly one. On the other hand, the higher frequency of data input reduces the fluctuation of RF estimation accuracy (i.e., the smallest SD with 4-hourly and biggest SD with the weekly frequency). In summary, for one nutrient, a higher sampling frequency typically causes the RF to yield a higher estimation accuracy.

**Table 4:** Results of the analysis-of-variance (ANOVA) test.

Nutrient	Statistical metric	Sampling frequency (n = 30)			F
		Weekly (Mean ± SD)	Daily (Mean ± SD)	Four-hourly (Mean ± SD)	
TP	*R* ^2^	0.602 ± 0.057 c	0.692 ± 0.026 b	0.785 ± 0.013 a	182.08**
	NSE	0.574 ± 0.042c	0.667 ± 0.026 b	0.781 ± 0.013 a	354.99**
TN	*R* ^2^	0.658 ± 0.065 c	0.761 ± 0.023 b	0.840 ± 0.009 a	150.21**
	NSE	0.639 ± 0.059 c	0.735 ± 0.024 b	0.837 ± 0.009 a	205.18**
NH_4_^+^-N	*R* ^2^	0.598 ± 0.062 c	0.676 ± 0.025 b	0.749 ± 0.013 a	107.29**
	NSE	0.559 ± 0.069 c	0.668 ± 0.025 b	0.747 ± 0.012 a	131.95**

Note: Statistical significance in the ANOVA test is denoted by asterisks for both *p* < 0.05 (*) and *p* < 0.01 (**). The F value denotes the ratio of the mean square between groups to the mean square within groups. The larger F value represents the larger difference between the groups. The different letters (a-c) after the numbers (Mean ± SD) indicated the significant differences between three sampling frequencies, while the same letters indicated that there are not significant differences.

### 3.4 Relative importance of input indicators

**Fig 7:**
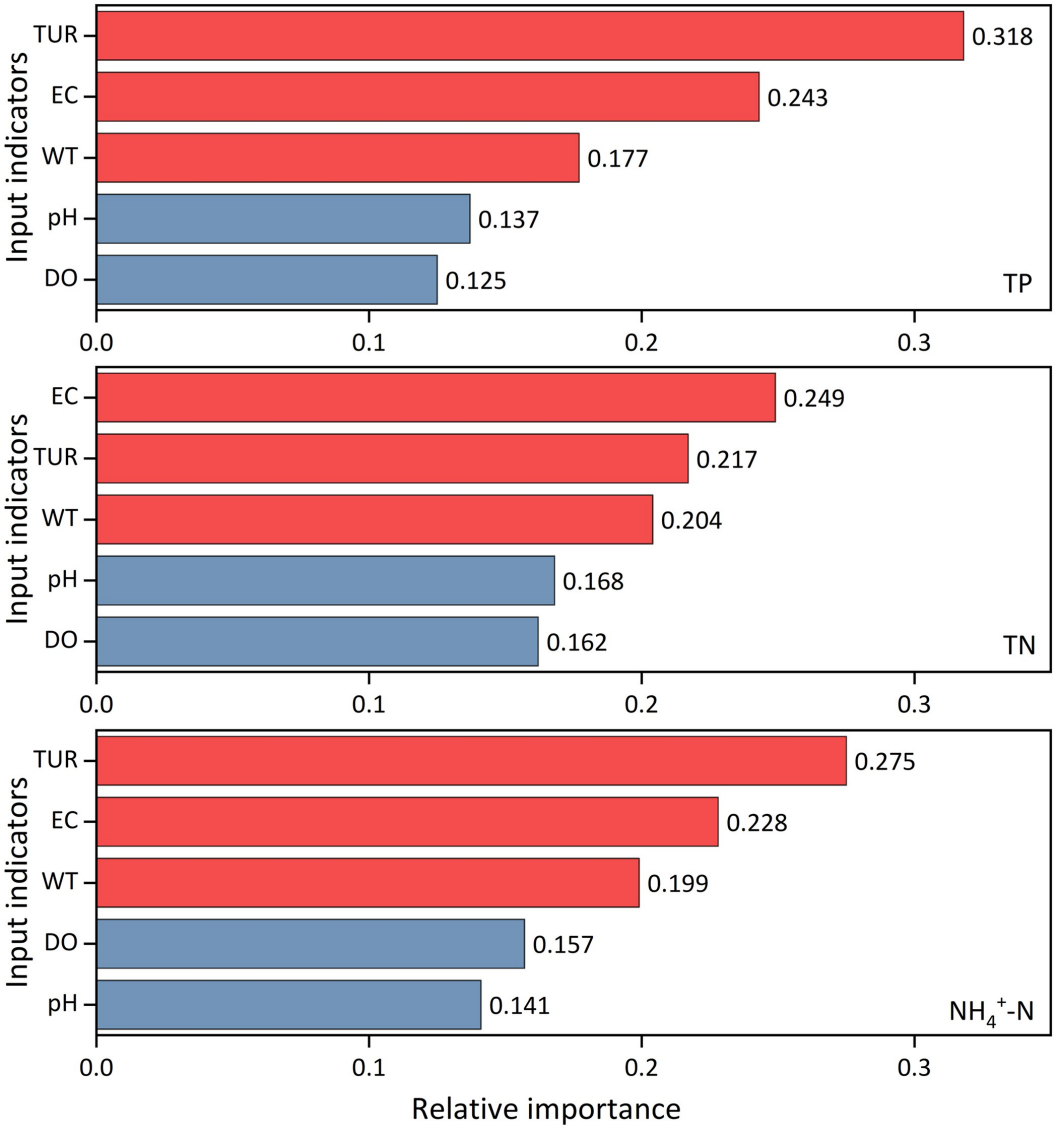
Relative importance analysis results of five input indicators in the random-forest (RF) model.

To clarify the relative importance of the five alternative inputs and find the key indicators in the nutrient concentration estimations, the RF with the 4-hourly sampling frequency scenario was used. As shown in Fig 7, EC, TUR, and WT are the three most important indicators. TUR shows the highest relative importance in controlling the estimation accuracy of TP and NH_4_^+^-N, while EC is the most important indicator for the estimation of TN. Comparatively, pH and DO are the two least important indicators of nutrient estimation. Based on the relative importance analysis, we found the three key indicators that affect the nutrient concentration dynamics among the five conventional water quality indicators.

## 4 Discussion

### 4.1 Uncertainty of model estimation

Machine learning models have large uncertainties associated with their unique structures, hyperparameter adjustment requirements, and data input [36,43]. The division rules of training and testing sets and the addition or deletion of input indicators can also cause fluctuations of estimation accuracy [44]. The same machine learning algorithm mentioned in different studies will perform differently due to the above-mentioned factors. Different machine learning algorithms will also perform differently even if be in the same study area and using the same dataset (specific information can be seen in the Table in S5 Text, Supporting information). There is no single algorithm that works best under all conditions. [45]. Firstly, we compared the estimation accuracy of three widely used machine learning models in our study area. In addition to the differences of the model, we controlled other variables to maintain consistency. The results of the testing step showed that the estimation accuracy of the RF model was the highest among the three models. The RF had the highest *R*^2^ and NSE values (*R*^2^ = 0.801, 0.859, and 0.759 for TP, TN, and NH_4_^+^-N; NSE = 0.785, 0.853, and 0.748 for these three nutrients) and the lowest RMSE and MAE values (RMSE = 0.039, 0.284, and 0.087 for TP, TN, and NH_4_^+^-N; MAE = 0.024, 0.189, and 0.057 for these three nutrients) (Table 2). The Taylor diagrams (Fig 4) also supported this finding. In these diagrams, the RF model was always the closest to the point represented by the observation, whereas the BPNN was the farthest from observation.

Many studies compared the performance of different models under the same conditions. Some of them reached the same conclusion as ours, that the RF model may be a more viable tool than other models for estimating water quality [31,32,46]. We also found that the estimation accuracy of the SVM was higher than BPNN, which is also found in other studies [47,48].

On the other hand, the number of input indicators affects the estimation accuracy of the machine learning model [49]. Attention should be paid to the overfitting caused by excessive types of input indicators [38,50]. Simultaneously, the difficulty of data acquisition must be considered [39,51]. For the simplicity and feasibility of the model, the input indicators must be at a sufficiently small scale to make estimations [52]. For the convenience of data acquisition, we only selected five water-quality parameters that can be measured easily *in situ*. Manual sampling and experiments or automatic sensor monitoring can be the method to obtain model input data, and the obtained data can be used as input indicators for subsequent nutrient concentration estimations according to the proposed methodology.

Different sampling frequencies influence estimation accuracy when using machine learning methods [31,53]. Generally speaking, the higher sampling frequency means that a larger amount of data can be obtained in the same time period, which will cause the machine learning model to use more data to improve its learning ability and obtain better estimation performance. Thomas et al. [54] found that the *R*^2^ for phytoplankton estimation decreased from 0.89 at a resolution of 4-hourly to 0.74 at a 1-month resolution. Our study also showed that a higher sampling frequency led to higher accuracy (Figs 5 and 6 and Tables 3 and 4). Moreover, high-frequency data input also plays an important role in improving the estimation performance of the mechanism model. Jiang et al. [55] used two frequencies data input and catchment hydrology model named HYPE to estimate nitrate and evaluate uncertainty. They found that HYPE model better captured nitrate dynamics when using daily data than fortnightly data, and daily data produced smaller predictive uncertainty. However, Liu and Lu [56] compared the estimation accuracies of TP and TN concentration by the SVM and artificial neural network (ANN) models under monthly, bimonthly, and trimonthly sampling frequencies from January, 2005, to December, 2010. And they drew a different conclusion: a higher sampling frequency sometimes does not lead to improvements of estimation accuracy, which may even cause accuracy degradation (for example, using SVM and ANN to estimate the concentration of TP and TN under different sampling frequencies, the order of accuracy was that bimonthly > trimonthly > monthly). Their conclusions indicated that increasing the sampling frequency does not necessarily increase the estimation accuracy though the sampling frequency they selected was not the “high frequency”.

To evaluate the model performance due to sampling frequency, we used the high-frequency dataset to construct different sampling frequency scenarios, and we analyzed the changes in estimation accuracy. The ANOVA test showed that the mean accuracy of 30 replicate estimations with the 4-hourly sampling frequency data input (*R*^2^ = 0.785, NSE = 0.781 for TP; *R*^2^ = 0.840, NSE = 0.837 for TN; *R*^2^ = 0.749, NSE = 0.747 for NH_4_^+^-N) was significantly higher than that of the daily (*R*^2^ = 0.692, NSE = 0.667 for TP; *R*^2^ = 0.761, NSE = 0.735 for TN; *R*^2^ = 0.676, NSE = 0.668 for NH_4_^+^-N) and weekly (*R*^2^ = 0.602, NSE = 0.574 for TP; *R*^2^ = 0.658, NSE = 0.639 for TN; *R*^2^ = 0.598, NSE = 0.559 for NH_4_^+^-N) data (Table 4). One reason for this may be that more data inputs can lead to a better understanding of hidden patterns [57]. Alternatively, the 4-hourly frequency may better represent the actual situation (e.g., concentration mutations) than the daily and weekly frequencies. This indicates that when other conditions are consistent, the larger number of data input could help the model better reflect the patterns of change in the values estimated, leading to higher performance [58,59]. With the development of technology, high-frequency water-quality monitoring equipment are deployed to rivers worldwide, which helps society better grasp the water-quality change information needed to complete model simulations more accurately [6,60]. This ideal situation cannot be easily realized with low-frequency sampling methods and laboratory experiment. Therefore, we strongly recommend using high-frequency data to develop the RF model to grasp the dynamic changes of riverine nutrient concentration.

### 4.2 Different estimation accuracies among three nutrient concentrations

In this study, the RF model showed the highest estimation accuracy for TN and the lowest estimation accuracy for NH_4_^+^-N. During the period from January 2019 to March 2021, the CV of TN was the lowest, whereas that of NH_4_^+^-N was the highest (Table 1), which is consistent with the ranked estimation accuracy of the three nutrients. Owing to its active chemical properties, NH_4_^+^-N can be easily converted to nitrites and nitrates [61]. The data used in this study were collected using an automatic monitoring sensor located at the outlet of the watershed. Point-source emissions might lead to a sudden increase of nutrient concentrations in a short time, owing to rapid urbanization [62]. These factors make the variation in riverine nutrient concentrations larger and more difficult to estimate [60], especially for NH_4_^+^-N. We identified three key indicators (WT, EC and TUR) through the relative importance analysis in Section 3.4. They have always been the top three important in the estimation of TP, TN and NH_4_^+^-N concentrations. Interestingly, except TUR, there are only weak correlations between WT as well as EC and nutrients. These indicated that WT, EC and TUR have a great impact on the modeling of nutrient concentration dynamics, and the importance could not be fully reflected in the results of correlation analysis. In future research, we may verify our findings above by using different combinations of input indicators. Also, we may evaluate the changes of model estimation accuracy by leaving out relatively less important indicator (such as pH or DO) to develop a more simplified model with minimal impact on model accuracy.

The RF model underestimated higher concentrations. This underestimation occurs frequently when using a machine learning algorithm to estimate numerous variables [4,19,57,60,63]. There are several reasons leading to the model underestimation of the peak nutrient concentration: the occasionally unusual observations or the fact that the five inputs selected for this study did not fully include the indicators affecting nutrient concentrations. Or some peaks were mistakenly removed as outliers when performing the outlier elimination operation.

### 4.3 Limitations and future agenda

Notwithstanding the success of machine learning in water-quality estimations, some limitations continue to hamper its wider use and impact. One limitation is the model interpretability [64]. Although machine learning models can fit observations well, it is difficult to trace their mechanism of temporal and spatial changes. The main purpose of this study was to develop a regression model that could accurately estimate nutrient concentrations; hence, the physical mechanism of nutrient changes was omitted. We instead explored the uncertainty induced by the sampling frequencies. Therefore, the uncertainties caused by different models were briefly evaluated and without cross-validation. Furthermore, there was only one automatic monitor at the outlet of the watershed studied. Thus, we used the so far water quality indicators only from one location for modelling and analysis. This may not sufficiently reflect all hydrological processes in the watershed.

Considering the continuous implementation of the follow-up work in our study area, this study only used five easily available indicators as data input, which eliminated the need for laboratory experiments. The input indicators can be obtained by sampling and measuring using a portable water-quality monitor along rivers and creeks, or by the sensor located in the outlet of the watershed. However, the convenience of the proposed methodology means that some important physical and chemical parameters (i.e., precipitation, flow, point source discharge, non-point source pollution, some water quality parameters, etc.) that affect the changes of nutrient concentrations were discarded. This is an inevitable problem due to the scarcity of data and the inconsistent time resolution of data from different sources. In subsequent work, we may consider adding more parameters related to the process mechanism as the input data to enhance the interpretability of the machine learning models. In addition, the good estimation results of this study were realized by the excellent fitting ability of machine learning algorithms and high-frequency data. In the future, the model should be continuously optimized or coupled with data-denoising algorithms, such as wavelet transforms, for performance improvement.

## 5 Conclusions

We developed the RF model to estimate the concentrations of TP, TN, and NH_4_^+^-N using only five easily obtainable water-quality indicators (i.e., WT, pH, EC, DO, and TUR) as surrogates. We built SVM and BPNN models for comparison to RF, and the results showed that RF performed best. We evaluated the estimation uncertainties related to the sampling frequencies (i.e., 4-hourly, daily, and weekly). There was a significant improvement of model accuracy when the frequency of data input was increased. When using the 4-hourly sampling frequency dataset, RF explained the dynamic variation in TP (79 ± 1.3%), TN (84 ± 0.9%), and NH_4_^+^-N (75 ± 1.3%). We attribute the accurate estimation of nutrient concentrations to the availability of high-frequency monitoring data, which has shown great potential in water-quality indicator estimations that cannot otherwise be easily realized by daily/weekly sampling routines. Furthermore, EC, TUR, and WT were identified as the key indicators to the estimation of TP, TN, and NH_4_^+^-N. The RF model is an effective alternative for estimating riverine nutrient concentrations when using high sampling frequency data, which is essential for sustainable water management in watersheds producing scarce water-quality data.

## Supporting information

S1 Text Monitoring sensors for different water quality parameters. (DOCX)

S2 Text Selection of hyperparameters for random forest. (DOCX)

S3 Text Selection of hyperparameters for Back propagation neural network. (DOCX)

S4 Text Selection of hyperparameters for Support vector machine. (DOCX)

S5 Text Comparing the performance of our study with other water quality estimation works using different machine learning models. (DOCX)

S6 Text Water quality datasets with different sampling frequencies. (XLSX)
