# Supplementary material for: Machine learning-based estimation of riverine nutrient concentrations and associated uncertainties caused by sampling frequencies
Source: PLoS One. 2022 Jul 13;17(7):e0271458. doi: 10.1371/journal.pone.0271458 (PMC9278742; doi:10.1371/journal.pone.0271458)
Supplement: S1 Text — (DOCX) [file pone.0271458.s001.docx]

# Monitoring sensors for different water quality parameters

water temperature (WT), hydrogen ion concentration (pH), electrical conductivity (EC), dissolved oxygen (DO), and turbidity (TUR): EST-WQMS, China

total phosphorus (TP): EST-2003, China

total nitrogen (TN): EST-ZHYQ3362, China

ammonia nitrogen (NH4+-N): EST-2004, China
