# Supplementary material for: Machine learning-based estimation of riverine nutrient concentrations and associated uncertainties caused by sampling frequencies
Source: PLoS One. 2022 Jul 13;17(7):e0271458. doi: 10.1371/journal.pone.0271458 (PMC9278742; doi:10.1371/journal.pone.0271458)
Supplement: S2 Text — (DOCX) [file pone.0271458.s002.docx]

## Selection of hyperparameters for random forest

Most of the hyperparameters in the RF model we used were default parameters, and only the ntrees (the number of decision trees) and min-leaf (the minimum number of samples contained at the leaf nodes) in the RF model were adjusted. First, ntrees was set to 300. It can be observed from S2 Fig. that when this parameter was higher than 100, the smallest out-of-bag error value was reduced insignificantly. Considering the efficiency of model calculations, setting this parameter value to 100 could guarantee good estimation results. For min-leaf, we tested five values (1, 5, 10, 20, and 50) and found that the best estimation accuracy in cross-validation step could be obtained when the value was 1.


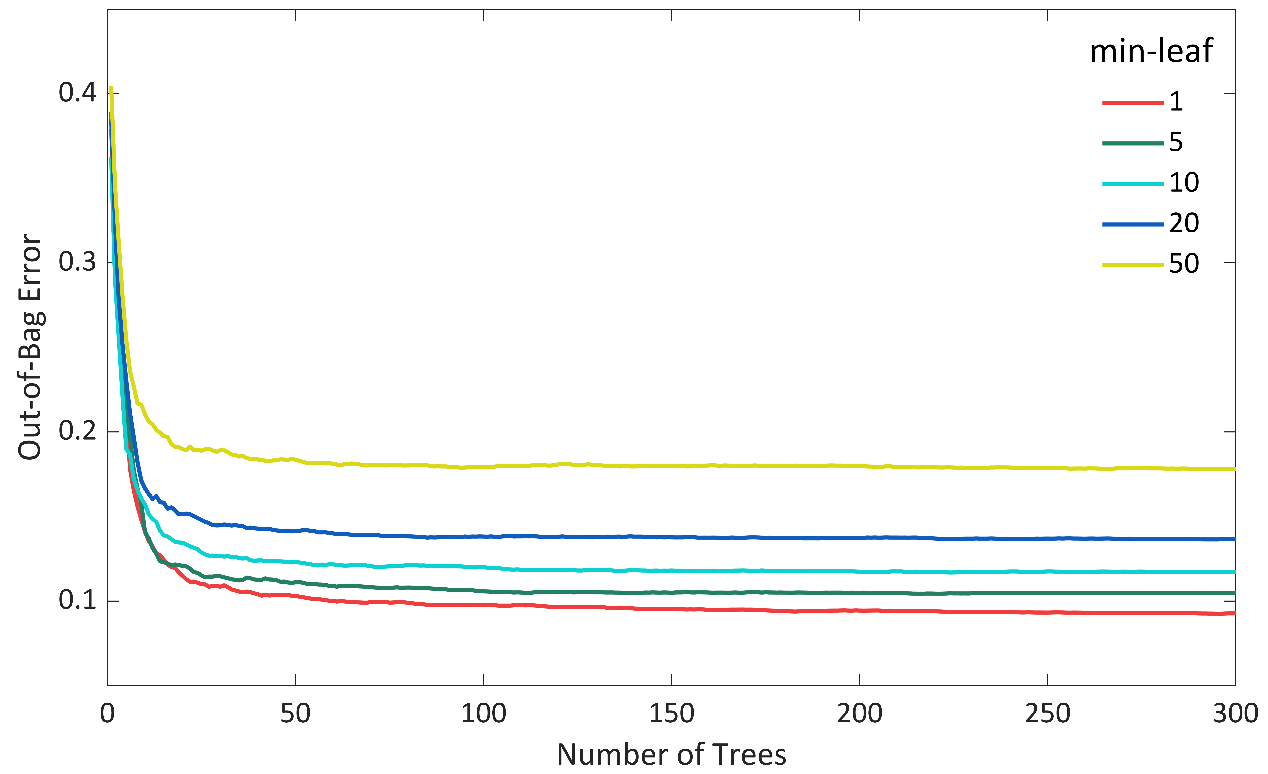


**S2 Fig. Relationship between the number of trees and out-of-bag error under different values of min-leaf**
