# Supplementary material for: Machine learning-based estimation of riverine nutrient concentrations and associated uncertainties caused by sampling frequencies
Source: PLoS One. 2022 Jul 13;17(7):e0271458. doi: 10.1371/journal.pone.0271458 (PMC9278742; doi:10.1371/journal.pone.0271458)
Supplement: S3 Text — (DOCX) [file pone.0271458.s003.docx]

# Selection of hyperparameters for Back propagation neural network

The most commonly used three-layer neural network was used, that is, the form of input layer - hidden layer - output layer. The initial default parameters included momentum, learning rule, learning coefficient ratio, and the convergence criterion (training target). The momentum method was used to adjust the weight and deviation repeatedly in the training process to minimize the network error. The network is very sensitive to the number of nodes in its hidden layer, too few nodes would lead to insufficient fitting, and too many nodes would lead to excessive fitting. To achieve the best number of hidden nodes, the following empirical formula was used to test the number of 3 to 13 hidden nodes, and the neural network with each number of nodes was tested 15 times.

$m=\sqrt{n+l}+c$ (1)

Among them, m is the number of hidden layer nodes, n is the number of input layer nodes, *l* is the number of output layer nodes, and c is a constant between 1-10.

For TP, TN, and NH_4_^+^-N, the estimation results of validation set for BPNN with different numbers of hidden nodes are shown in Fig. S2. It could be seen that the network with thirteen hidden nodes did not produce the highest *R*^2^. According to the results in S3 Fig., the nine and ten hidden nodes were the best choices. Therefore, the number of hidden nodes was set to nine to estimate the concentration of TP, and ten to estimate the concentration of TN and NH_4_^+^-N.


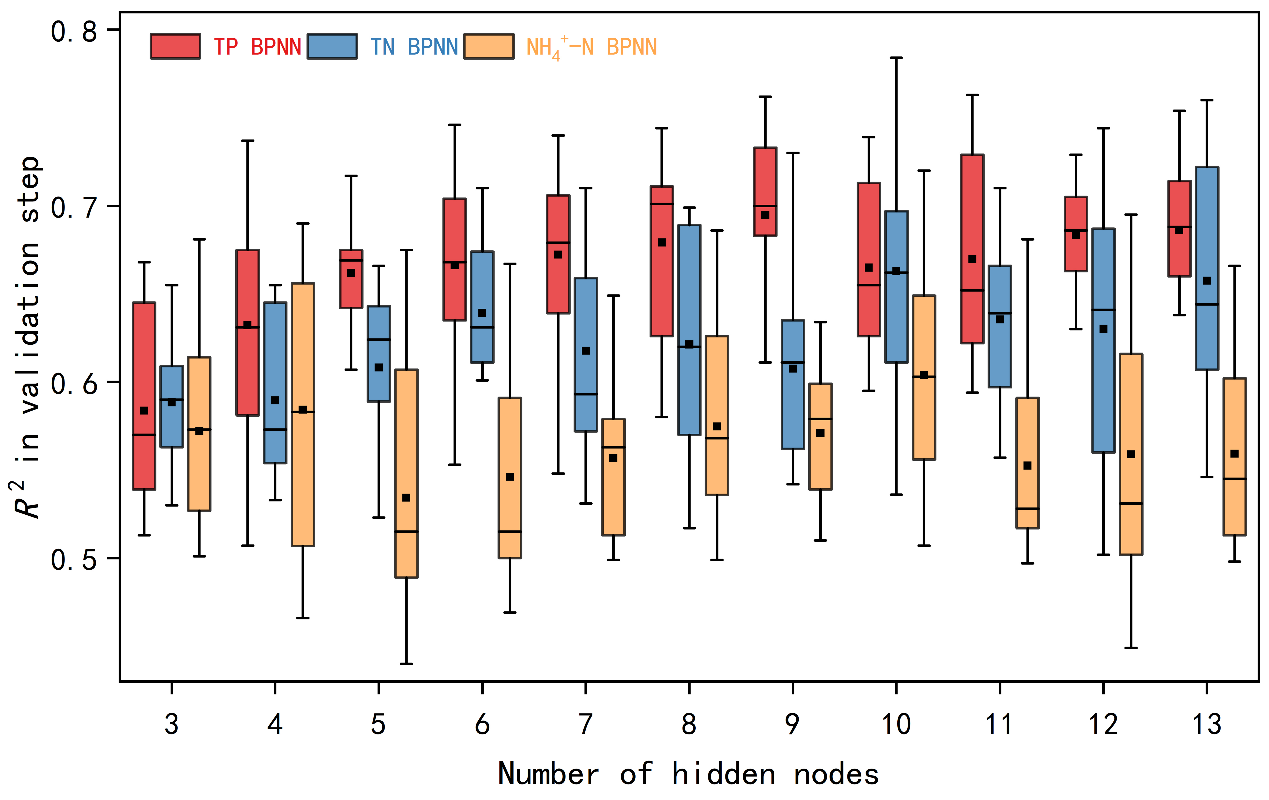


**S3 Fig. Model performances in validation step of BPNN with different numbers of hidden nodes for TP, TN, and NH_4_^+^-N**
