# Supplementary material for: Machine learning-based estimation of riverine nutrient concentrations and associated uncertainties caused by sampling frequencies
Source: PLoS One. 2022 Jul 13;17(7):e0271458. doi: 10.1371/journal.pone.0271458 (PMC9278742; doi:10.1371/journal.pone.0271458)
Supplement: S4 Text — (DOCX) [file pone.0271458.s004.docx]

## Selection of hyperparameters for Support vector machine

The RBF (Radial Basis Function Kernel) was selected as the kernel function, which is also called radial basis function. The range of the initial hyperparameters c1 and g1 in the SVM model was limited to [-10, 10, 0.5] through the Grid search method, that is, the upper and lower limits were set to 10 and -10, and the resolution was 0.5. Then the hyperparameters were automatically optimized by exhaustive means. The optimal c/g is equal to 2 to the c1/g1 power. Where c is the penalty coefficient, and g (gamma) is a hyperparameter after choosing RBF as the kernel. Then, the SVM model was trained by using the selected optimal hyperparameters. The testing data set was used to verify the accuracy of the SVM model. The specific hyperparameter values are shown in S4 Table.

**S4 Table. Optimal hyperparameters of the SVM model selected by grid search**

| Nutrients | Optimal parameters | |
| --- | --- | --- |
|  | c | g |
| TP | 1.414 | 8.001 |
| TN | 0.707 | 6.001 |
| NH_4_^+^-N | 0.500 | 1.414 |
