# Supplementary material for: Machine learning-based estimation of riverine nutrient concentrations and associated uncertainties caused by sampling frequencies
Source: PLoS One. 2022 Jul 13;17(7):e0271458. doi: 10.1371/journal.pone.0271458 (PMC9278742; doi:10.1371/journal.pone.0271458)
Supplement: S5 Text — (DOCX) [file pone.0271458.s005.docx]

**S5 Table. Comparing the performance of our study with other water quality estimation works using different machine learning models.**

| Researches | Estimated object | Model selection | Data frequency | Types of input indicators | Input indicators | Accuracy |
| --- | --- | --- | --- | --- | --- | --- |
| (Asadollah et al., 2021) | WQI | SVM | Monthly | 10 | EC, pH, WT, TUR, DO, BOD, COD, NO_3_-N, NO_2_-N, PO_4_^3-^ | *R*^2^ = 0.967 |
| (Csábrági et al., 2017) | DO | GRNN | Fortnightly | 4 | pH, RF, WT, EC, | *R*^2^ = 0.720 |
| (Deng et al., 2021) | Chl-a | SVM | Weekly | 8 | Chl-a, BOD, TIN, DO, pH, PO_4_^3-^, SDD, WT | *R* = 0.984 |
| (Harrison et al., 2021) | TN  TP | RF | 1-15 min | / | Time, soil moisture, hydrostatic pressure, WT, TUR, EC, pH, FDOM, variables with time lags | NSE=0.85  NSE=0.74 |
| (Leong et al., 2019) | WQI | SVM  LS-SVM | / | 6 | DO, BOD, COD, TSS, NH_4_^+^-N, pH | *R*^2^ = 0.918  *R*^2^ = 0.923 |
| (Liu and Lu, 2014) | TP | ANN | Bimonthly  Monthly | 8  8 | DO, NO_3_^-^N, NH_4_^+^-N, CODmn, WT, EC, pH, TUR | *R*^2^ = 0.841  *R*^2^ = 0.743 |
| (Lu and Ma, 2020) | FDOM | XGBoost  RF | Daily | 6 | The value of each estimated object in the previous 6 days | RMSE = 0.33  RMSE = 1.37 |
| (Mamun et al., 2019) | Chl-a | SVM | Monthly | 9 | precipitation, DO, BOD, COD, TSS, TN, TP, NP ratios, WT, EC | *R*^2^ = 0.810 |
| (Ren et al., 2020) | DO | DBN | 10 min | 6 | DO, pH, WT, TUR, Liquid level, NH_4_^+^-N | *R*^2^= 0.936 |
| (Wen et al., 2013) | DO | ANN | Monthly | 8 | pH, EC, Cl^−^, Ca^2+^, TA, TH, NO_3_^-^N, NH_4_^+^-N | *R* = 0.968 |
| This study | TP  TN | RF | 4-hourly | 5 | WT, pH, EC, DO, TUR | *R*^2^ = 0.785  *R*^2^ = 0.840 |
|  | NH_4_^+^-N |  |  |  |  | *R*^2^ = 0.749 |

Abbreviations: WQI water quality index, SVM support vector machine, EC electrical conductivity, WT water temperature, TUR turbidity, DO dissolved oxygen, BOD biochemical oxygen demand, COD chemical oxygen demand, NO3-N nitrate nitrogen, NO2-N nitrite nitrogen, PO_4_^3-^ phosphate radical, GRNN general regression neural network, pH hydrogen ion concentration, RF runoff, Chl-a Chlorophyll a, TIN total inorganic nitrogen, SDD the secchi-disc depth, TN total nitrogen, TP total phosphorus, RF random forest, FDOM fluorescent dissolved organic matter, LS-SVM least square-support vector machine, TSS total suspended solids, NH_4_^+^-N ammonia nitrogen, ANN Artificial Neural Network, CODmn permanganate index, XGBoost extreme gradient boosting, DBN Deep Belief Network, Cl^−^ Chloride ion, Ca^2+^ calcium, TA total alkalinity, TH total hardness, *R*^2^ the coefficient of determination, R correlation coefficient, NSE Nash-Sutcliffe efficiency, RMSE root mean squared error

# S3 Table References

Asadollah SBHS, Sharafati A, Motta D, Yaseen ZM. River water quality index prediction and uncertainty analysis: A comparative study of machine learning models. Journal of Environmental Chemical Engineering 2021; 9.

Csábrági A, Molnár S, Tanos P, Kovács J. Application of artificial neural networks to the forecasting of dissolved oxygen content in the Hungarian section of the river Danube. Ecological Engineering 2017; 100: 63-72.

Deng T, Chau K-W, Duan H-F. Machine learning based marine water quality prediction for coastal hydro-environment management. Journal of Environmental Management 2021; 284.

Harrison JW, Lucius MA, Farrell JL, Eichler LW, Relyea RA. Prediction of stream nitrogen and phosphorus concentrations from high-frequency sensors using Random Forests Regression. Sci Total Environ 2021; 763: 143005.

Leong WC, Bahadori A, Zhang J, Ahmad Z. Prediction of water quality index (WQI) using support vector machine (SVM) and least square-support vector machine (LS-SVM). International Journal of River Basin Management 2019: 1-8.

Liu M, Lu J. Support vector machine-an alternative to artificial neuron network for water quality forecasting in an agricultural nonpoint source polluted river? Environ Sci Pollut Res Int 2014; 21: 11036-53.

Lu H, Ma X. Hybrid decision tree-based machine learning models for short-term water quality prediction. Chemosphere 2020; 249: 126169.

Mamun M, Kim J-J, Alam MA, An K-G. Prediction of Algal Chlorophyll-a and Water Clarity in Monsoon-Region Reservoir Using Machine Learning Approaches. Water 2019; 12.

Ren Q, Wang X, Li W, Wei Y, An D. Research of dissolved oxygen prediction in recirculating aquaculture systems based on deep belief network. Aquacultural Engineering 2020; 90.

Wen X, Fang J, Diao M, Zhang C. Artificial neural network modeling of dissolved oxygen in the Heihe River, Northwestern China. Environ Monit Assess 2013; 185: 4361-71.
